# Signaling Pathways Governing Cardiomyocyte Differentiation

**DOI:** 10.3390/genes15060798

**Published:** 2024-06-18

**Authors:** Isaiah K. Mensah, Humaira Gowher

**Affiliations:** Department of Biochemistry, Purdue University, West Lafayette, IN 47907, USA

**Keywords:** heart, cardiomyocytes, signaling, iPSCs, ESCs, BMP, Notch, sonic hedgehog, Hippo, Wnt signaling

## Abstract

Cardiomyocytes are the largest cell type that make up the heart and confer beating activity to the heart. The proper differentiation of cardiomyocytes relies on the efficient transmission and perception of differentiation cues from several signaling pathways that influence cardiomyocyte-specific gene expression programs. Signaling pathways also mediate intercellular communications to promote proper cardiomyocyte differentiation. We have reviewed the major signaling pathways involved in cardiomyocyte differentiation, including the BMP, Notch, sonic hedgehog, Hippo, and Wnt signaling pathways. Additionally, we highlight the differences between different cardiomyocyte cell lines and the use of these signaling pathways in the differentiation of cardiomyocytes from stem cells. Finally, we conclude by discussing open questions and current gaps in knowledge about the in vitro differentiation of cardiomyocytes and propose new avenues of research to fill those gaps.

## 1. Introduction

Cardiomyocytes (CMs) are terminally differentiated cells that originate from the mesoderm during early embryogenesis and make up a majority of the heart tissue [1,2,3]. In mammals, a subset of mesodermal cells generates the first heart field (FHF) and the second heart field (SHF), which are two distinct main reservoirs for CMs [4,5]. The cardiomyocytes originating from the FHF differentiate to form the bulk of the primitive heart tissue in the early embryo. As the embryo grows, the cells from the SHF differentiate and contribute cardiomyocytes to the developing heart, which circulates oxygen and nutrients to other parts of the embryo [6]. Signaling pathways orchestrate the differentiation of cardiomyocytes during early developmental stages [7]. Most signaling events are either autocrine, where the cells secrete signaling molecules that bind to their receptors, or paracrine, where cells release signaling molecules that bind to receptors of other neighboring cells. The signaling cues eventually influence gene expression and determine the transcriptional state of cells as they transition from pluripotent cells into mesodermal cells and become cardiomyocytes during development. The main signaling pathways that regulate cardiomyocyte differentiation include the bone morphogenetic protein (BMP) pathway, the fibroblast growth factor (FGF) pathway, the Notch pathway, the sonic hedgehog pathway, Hippo signaling, and the Wnt pathway. In addition to the specificity of the temporal activity of signaling pathways, some pathways function independently, and others act in concert to regulate cardiomyocyte differentiation.

Primary cardiomyocytes can be isolated from the heart and maintained in vitro under specific culture conditions for therapeutic drug monitoring. However, primary cardiomyocytes cannot proliferate in tissue culture and are inadequate for studying transcriptional processes that regulate cardiomyocyte differentiation. To circumvent these issues, cardiomyocytes can be generated from embryonic stem cells (ESCs) and induced pluripotent stem cells (iPSCs), which provide several advantages, such as ease of genetic manipulation in studying early and specific stages of cardiomyocyte differentiation. In this review, we have compiled current studies on the signaling pathways and provide comprehensive insights into in vitro models of cardiomyocyte differentiation. The holistic understanding of signaling pathways and their utilization for the culture and generation of cardiomyocytes holds substantial promise for therapeutic purposes.

### 1.1. The BMP Signaling Pathway

**Figure 1:**
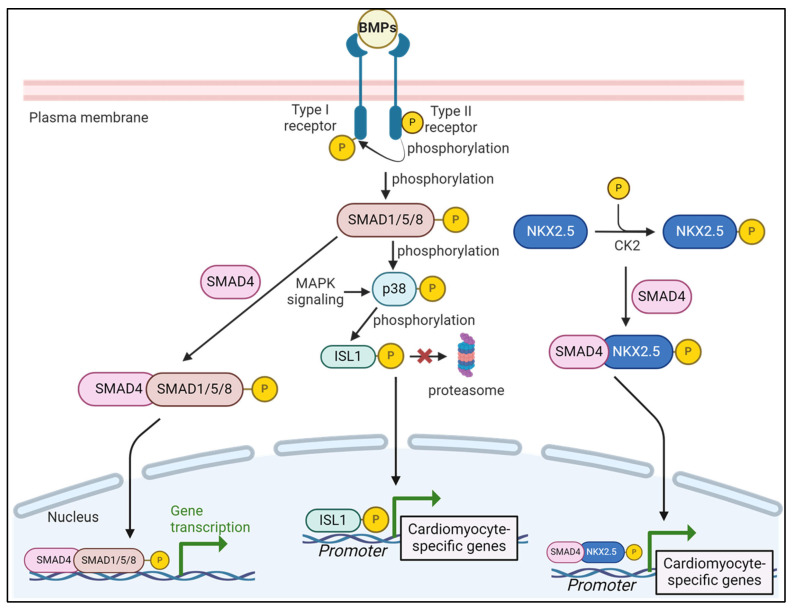
The BMP pathway in cardiomyocyte differentiation. The BMP signaling cascade begins with the binding of BMP ligands to type I and II transmembrane receptors. Phosphorylation of the receptors leads to the phosphorylation of SMADs, which then interact with SMAD4 and translocate into the nucleus to promote gene expression. Phosphorylated SMADs also phosphorylate p38 from the MAPK signaling pathway, which in turn phosphorylate ISL1 and prevent proteasomal degradation of ISL1, resulting in ISL1 translocation into the nucleus to turn on cardiomyocyte-specific genes. Additionally, Casein kinase 2 (CK2) phosphorylates, NKX2.5, and the phosphorylated active NKX2.5 interacts with SMAD4 to translocate NKX2.5 into the nucleus to turn on cardiomyocyte-specific genes. Created with BioRender.com.

Bone morphogenic proteins (BMPs) derive their name from initial discoveries showing their role in inducing bone formation [8]. The BMPs comprise about 20 proteins and are members of the TGF-β superfamily. Briefly, the BMP pathway begins with the secretion of BMPs as glycosylated homo- and heterodimers that form a heteromeric complex with type I and type II receptors on the plasma membrane of cells [9]. On the intracellular side of the plasma membrane, the heteromeric complex formation brings the type II receptors near the type I receptor to phosphorylate and activate the type I receptor in the kinase domain, leading to a cascade of phosphorylation events that phosphorylate SMAD1, SMAD5, or SMAD8. The phosphorylated SMADs form a complex with SMAD4, which then translocates and accumulates in the nucleus to elicit transcriptional changes in gene expression (Figure 1).

BMP signaling is antagonistic to pluripotency and promotes differentiation during development [10]. Additionally, the BMP signaling pathway promotes the differentiation of mesoderm cells into cardiac lineages. The cardiac commitment is achieved by the simultaneous expression of BMP4 and BMP7 from the ectoderm and BMP5 and BMP2 from the endoderm onto the neighboring mesoderm cells [11,12]. In chick embryos, studies have shown that BMPs and other factors are expressed in the lateral plate endoderm as cardiogenic paracrine factors to promote cardiomyocyte differentiation from mesoderm cells [13]. The significance of BMP signaling in cardiac development is further supported by the embryonic lethality of BMP2-deficient mice with severe cardiac defects [14]. During mesoderm specification into cardiomyocytes, the extracellular BMP signaling inhibitors, Noggin (NOG), Chordin (CHRD), and Follistatin (FST), are expressed [15,16,17]. However, their direct involvement in cardiomyocyte differentiation is unclear. The disruption of either BMP inhibitor or CHRD and NOG double knockout shows no cardiac defects during development [17,18]. Yet, the homozygous knockout of CHRD in mice causes early lethality, and surviving mice display signs of DiGeorge Syndrome, a congenital disorder that causes abnormalities in the heart. It is possible that BMP signaling inhibitors have redundant roles. Therefore, a triple knockout study may uncover their direct role in cardiomyocyte differentiation during development. 

BMP signaling promotes OFT myocardial development by regulating the miRNA 17-92 cluster through a conserved Smad binding element, identifying a novel BMP-miRNA pathway during cardiac development [19]. Additionally, BMP signaling regulates the expression and function of core transcription factors involved in cardiomyocyte differentiation, such as Hand1, Isl1, and Nkx2.5. The loss-of-function and gain-of-function mutant embryos decrease and increase the expression of Hand1, respectively [20]. Mechanistically, SMADs directly bind to Hand1 regulator elements to induce their expression during cardiomyocyte differentiation [20]. Furthermore, via the interaction of SMAD4 and NKX2.5, BMP signaling promotes the nuclear localization of NKX2.5 [11,21] (Figure 1). A crosstalk between BMP signaling and MAPK signaling through the p38-mediated phosphorylation of ISL1 prevents ISL1 proteasomal degradation during cardiomyocyte differentiation [22] (Figure 1). Studies indicate that BMP signaling has opposite effects on cardiac differentiation [23]. Using zebrafish as a model system revealed that BMP signaling remains active during differentiation in the anterior lateral plate mesoderm, a hub of cardiac progenitor cells, post-gastrulation. However, Smad6 inhibits the BMP signaling pathway as cardiac differentiation proceeds, which is required for proper cardiac development [23]. 

### 1.2. The FGF Signaling Pathway

The fibroblast growth factor (FGF) family comprises 22 secreted signaling proteins in humans and mice. These FGF proteins interact with transmembrane receptor tyrosine kinases and intracellular non-signaling proteins (iFGFs) [24]. Based on phylogenetic analysis, secreted FGFs are grouped into paracrine, endocrine, or intracrine FGFs [24,25]. The paracrine FGFs are further grouped into five subfamilies, while the endocrine and intracrine FGFs are each grouped into one subfamily [26,27,28,29,30,31]. The FGF signaling pathway initiates with the binding of paracrine FGFs to FGF receptors (FGFRs) located on the plasma membrane. The binding of FGFs to their receptors is mediated by heparin/heparin sulfate (HS) or heparin sulfate proteoglycans (HSPGs), which also prevent the diffusion of FGFs across the extracellular matrix [27,32]. Unlike paracrine FGFs, endocrine FGFs require protein cofactors such as αKlotho, βKlotho, or KLPH to mediate receptor binding [33,34]. The binding of FGFs to FGFRs induces a conformational change in the receptors to form a ternary FGF-FGFR-HS/HSPG complex, which phosphorylates and activates the intracellular domain of the FGFRs [35]. The activated FGFRs are coupled to other dominant intracellular signaling events such as RAS-MAPK, PI3K-AKT, PLCγ, and STAT signaling pathways [24,36]. 

The FGF signaling pathway has been shown to play several roles in cell differentiation and development [37,38]. During early development in Xenopus, FGFs have been shown to induce the formation and maintenance of mesoderm cell populations [39,40,41]. FGF-2 and FGF-9 are expressed maternally ahead of mesoderm induction. FGF-3 and FGF-4 are induced in the newly formed mesoderm following gastrulation, and the inhibition of FGF signaling significantly reduces mesoderm cell populations [39]. Similarly, FGF signaling promotes mesoderm formation in zebrafish developmental models [42]. In mouse development, Fgfr1 is expressed throughout the epiblast, and its expression later becomes localized along the primitive streak. The genetic ablation of Fgfr1 significantly reduces mesodermal populations and is embryonic lethal [43]. FGF signaling has also been shown to function with BMP signaling to promote mesoderm formation during development [44]. New studies suggest that the defects in mesoderm development observed in the absence of FGF signaling may be a consequence of attenuated WNT signaling [45].

Post mesoderm formation, FGF signaling is heavily involved in forming the second heart field, a primary source of cardiomyocytes [46]. The combination of FGF2 and BMP2 has also been shown to promote cardiomyocyte differentiation from stem cell models, highlighting the crosstalk between the BMP and FGF signaling pathways [47,48]. Similarly, FGF10, via its interaction with FGFRs, promotes cardiomyocyte differentiation [49]. A more recent study also described the role of FGF signaling through the AKT pathway in the regeneration and survival of cardiomyocytes in zebrafish. The loss of FGF signaling increased cardiomyocyte death and reduced AKT pathways in cardiomyocytes, further highlighting the requirement for the FGF signaling pathway during cardiomyocyte differentiation [50]. These studies suggest that FGF signaling may play biphasic roles in the differentiation of cardiomyocytes. Indeed, studies show that the activation of FGF signaling is required for cardiac mesoderm differentiation. However, continued activation inhibits further differentiation of mesoderm cells into cardiomyocytes. Mechanistically, FGF signaling inhibits FRS2α-mediated signals, activating autophagy, a self-digesting lysosomal-mediated process, promoting cardiomyocyte differentiation [51]. It is apparent that FGF signaling may interface with multiple biological processes to regulate cardiomyocyte development. It would be interesting to study the intersection of FGF signaling with multiple biological processes and pathways during cardiomyocyte differentiation. 

### 1.3. The Notch Signaling Pathway

The Notch pathway, although simple, has a unique framework of signaling events. The ligands (classified into Delta and Jagged families in mammals) and receptors of the Notch signaling pathway are transmembrane proteins consisting of epidermal growth factor (EGF)-like repeats [52,53]. The activation of the ligand and receptors of the Notch signaling pathway usually occurs through cell–cell contacts. The binding of the ligand on one cell to the receptor on another cell triggers two proteolytic events by the ADAM family metalloproteases and the γ-secretase to release the Notch intracellular domain (Notch-ICD) [52,54,55,56,57]. The Notch-ICD translocates into the nucleus, where it interacts with CSL (CBF1, Su(H), and LAG1), a DNA-binding protein, which recruits Mastermind (Mam), a coactivator of CSL, to promote gene transcription [52,58,59]. 

The Notch signaling pathway is essential in cardiomyocyte development [60]. Genetic ablation of the genes encoding either receptors or ligands of the Notch signaling pathway results in embryonic lethality due to defects in the development of the cardiovascular system [61,62,63,64,65]. There is a crosstalk between Notch signaling and BMP signaling (through BMP10) in the proliferation of cardiomyocytes [65,66]. For instance, the perturbation of Notch signaling attenuates the expression of BMP10 and is associated with reduced cardiomyocyte proliferation [65]. The earliest activated Notch-ICD was observed in the mesoderm, using antibodies against Notch-ICD for immunofluorescence at different developmental stages [64] and promoting the differentiation of cells into mesoderm post-gastrulation [67]. Additionally, whereas studies using Xenopus, zebrafish, and chicken also highlight the regulatory role of Notch signaling in the formation of the mesoderm [68,69], others show that the activation of Notch signaling in mesoderm increases the yield and efficiency of cardiomyocyte generation from hIPSc [70]. Interestingly, some studies have also shown that the inactivation of the Notch signaling pathway promotes the formation of mesoderm cells and the differentiation of the mesoderm into cardiomyocytes [69,71,72,73,74,75,76]. Future studies showing the compensatory or redundant role of other signaling pathways may shed more light on the mechanisms of Notch signaling during cardiomyocyte differentiation. 

### 1.4. The Sonic Hedgehog Signaling Pathway

The mammalian sonic hedgehog pathway consists of three ligands: sonic hedgehog (Shh), Indian hedgehog (Ihh), and Desert hedgehog (Dhh) [77]. Unlike other pathways, where the binding of ligands activates their receptors, the sonic hedgehog pathway is distinct in that ligand binding inactivates the receptor. Without the Shh glycoprotein ligand, the transmembrane protein Patched (Ptch1) receptor inhibits Smoothened (Smo), a seven-transmembrane GPCR-like receptor. The suppressor of Fused (SUFU) then sequesters GL1 (glioma-associated) in the cytoplasm to prevent the activation of GLI target genes [78,79,80]. However, the inhibition of Ptch1 by Shh impairs the inhibition of Smo, therefore abolishing the GL1-SUMO complex and allowing GL1 to translocate into the nucleus to turn on target genes. The sonic hedgehog signaling pathway is activated non-canonically, excluding GL1 transcription factors or Shh binding to the Ptch1 receptors [81]. 

The sonic hedgehog (Shh) pathway plays several roles in distinct lineage specifications, including cardiac development [82,83,84,85]. The tissue-specific depletion of Shh results in defects in cardiac outflow tract (OFT) separation and is required for signaling in myocardial cells derived from the anterior heart field (AHF) [86,87]. Mice lacking Smo, Shh, and Ihh show aberrant cardiac development, reduced cardiac size, and delayed expression of precardiac markers [88]. A recent study revealed that the deletion of GRK2/5/6, G-protein kinase receptors, disrupts Gli-mediated transcriptional activity, causing atrioventricular defects and embryonic lethality [89]. Interestingly, the deletion of one of the GRKs did not affect normal development, suggesting a redundant and compensatory mechanism of the GRKs during embryo development [89]. These data link GRKs in the modulation of the hedgehog signaling in fetal mouse hearts. The sonic hedgehog signaling also promotes cardiac development in zebrafish by functioning in a cell-autonomous manner, contributing to the myocardium [90]. The activation of sonic hedgehog signaling induces the aggregation of p19 cells and the expression of cardiac muscle genes [91,92]. Furthermore, Gli2 (glioma-associated factor 2), a transactivator of the Shh pathway, and MEF2C interact and complement each other to promote cardiomyocyte differentiation of P19 cells, linking Shh signaling with MEF2C in cardiac development [93]. Shh signaling via crosstalk with FGF signaling also promotes mesoderm patterning during mouse development [94]. More studies are needed to elucidate the mechanisms of sonic hedgehog signaling in regulating cardiomyocyte differentiation.

### 1.5. The Hippo Signaling Pathway

The Hippo signaling pathway is traditionally associated with the control of organ size during development [95]. In contrast to typical signaling pathways characterized by specific ligand-receptor interactions, the Hippo signaling pathway is modulated by a wide range of factors, including stress signals, cell density, soluble factors, cell polarity, and mechanical cues [96,97]. The mammalian Hippo pathway consists of the sterile 20-like protein kinase MST1/2, forming heterodimers with SAV1 through their C-terminal SARAH domains. This interaction is necessary for MST1/2 to phosphorylate SAV1, MOB1 (an ortholog of Mats), and LATS1/2 kinase (orthologs of Wts) [98,99]. The activated LATS1/2 then phosphorylates YAP (yes-associated protein) and TAZ (WW domain-containing transcription regulator protein 1), which causes 14-3-3 to interact with and sequester YAP/TAZ in the cytoplasm and inhibits their nuclear localization [100,101]. Additional phosphorylation of YAP/TAZ by casein kinase 1 triggers β-TrCP-mediated ubiquitination, leading to their subsequent proteasomal degradation [101,102,103]. The canonical interacting partners of YAP/TAZ in the nucleus are the TEAD family of transcription factors [104,105]. Without nuclear YAP/TAZ, TEAD functions as a default repressor by binding to VGLL4 (transcription cofactor vestigial-like protein 4) [106,107]. In the Hippo off state, the upstream kinases are inactive, leading to a dephosphorylated YAP/TAZ complex that translocates into the nucleus and associates with TEAD and other transcription factors to induce the transcription of target genes [108].

The Hippo signaling pathway is prevalent in early development and cardiomyocyte differentiation [109]. The double knockout of Yap1 and Wwtr1 in mice is lethal to embryos at the morula stage [110]. Embryonic stem cells lacking MST1/2 can differentiate into mesodermal lineages. However, further differentiation into cardiomyocytes is significantly impaired [111]. The activation of YAP1 in the Hippo pathway promotes cardiomyocyte proliferation and is implicated in cardiac regeneration following myocardial injury [112,113,114]. The overexpression of YAP1 extends neonatal cardiomyocyte proliferation [113], whereas the depletion of YAP1 impedes neonatal cardiomyocyte regeneration [114]. Interestingly, YAP1 inhibits the differentiation of stem cells into mesoderm lineages through the repression of Wnt-Activin-coregulated mesendoderm genes [115]. The inhibition of cardiomyocyte differentiation was further shown to be mediated via Wnt3 repression by YAP1 [116]. The activation of YAP1 promotes cardiomyocyte regeneration following cardiac injury. However, long-term activation results in cardiomyocyte dedifferentiation and heart failure in the presence of pressure overload (PO) [117]. Additionally, mice lacking Lats2, Salv, or Mst1/2 exhibited elevated cardiomyocyte proliferation, albeit by inhibiting WNT signaling [118]. These studies suggest an indirect and complicated role of Hippo signaling in cardiomyocyte differentiation. More studies are required to tease apart the activation versus inhibition of Hippo signaling in cardiomyocyte differentiation. 

### 1.6. The Wnt Signaling Pathway

**Figure 2:**
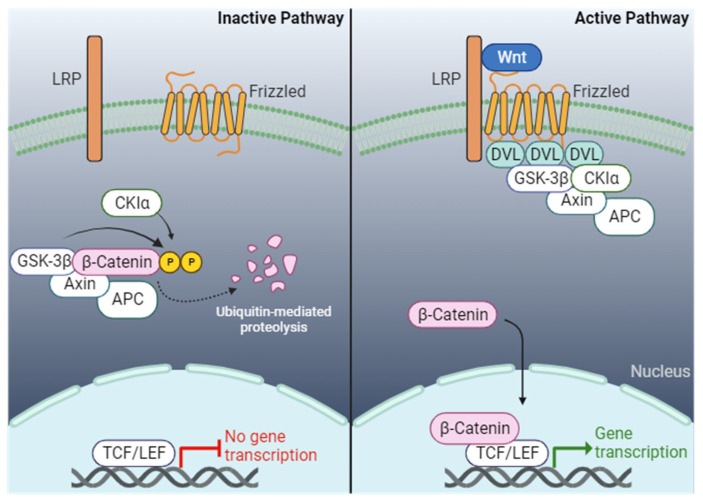
The WNT signaling pathway. Illustration of the WNT signaling pathway in the on and off state. When the WNT signaling pathway is inactive, the destruction complex consisting of Gsk3β, Axin, and APC phosphorylates β-catenin, leading to ubiquitination of β-catenin and eventual proteasomal degradation by the proteasome. In the active pathway, WNT ligands bind to the transmembrane frizzled receptor and LRP, which then sequester the destruction complex away from phosphorylating β-catenin. This allows β-catenin to translocate into the nucleus, interacting with TCF/LEF proteins to activate the expression of WNT target genes. Created with BioRender.com.

The Wnt/β-catenin pathway is a well-studied and evolutionary conserved signaling pathway in cell proliferation and development [119]. The canonical Wnt/β-catenin begins with the autocrine or paracrine binding of secreted Wnt glycoprotein ligands that bind to the Wnt receptor Frizzled (FZD; a seven-fold transmembrane protein) and the co-receptor LRP5/6 (low-density lipoprotein-related protein 5/6) located across the plasma membrane [120,121,122,123,124]. The binding of Wnt ligands to the receptors leads to the phosphorylation of LRP5/6, which transduces the signal to the cytoplasmic protein Disheveled (DVL) (Figure 2). This leads to the recruitment and oligomerization of DVL at the plasma membrane [125]. Consequently, the destruction complex consisting of APC (adenomatous polyposis coli), AXIN (axis inhibition protein), CK1 (casein kinase 1), and GSK3β (glycogen synthase-3-β) is recruited to associate with the Wnt receptors and DVL, which inhibits the phosphorylation of β-catenin by GSK3β, thereby preventing the proteasomal degradation of β-catenin. The cytoplasmic β-catenin levels increase, resulting in translocation into the nucleus where β-catenin interacts with TCF/LEF (T cell factor/Lymphoid enhancer-binding factor) family of transcription factors to activate Wnt target genes that mediate myriad phenotypes [126,127]. In the absence of a Wnt ligand, the FZD and LRP5/6 are separated, freeing the destruction complex to phosphorylate β-catenin at the N-terminal serine and threonine residues, leading to ubiquitination and eventual proteasomal degradation [126,128].

The Wnt/β-catenin plays a pivotal role in developing cardiac mesoderm and cardiomyocytes during early development [129]. Until recently, the role of Wnt/β-catenin in cardiomyocyte development was contrasting during development. For instance, the activation of Wnt signaling was shown to promote cardiac mesoderm formation and cardiomyocyte differentiation in Drosophila [130]. However, inhibiting the Wnt/β-catenin in the mesoderm of chicken and Xenopus embryos impairs cardiomyocyte differentiation [131,132]. Furthermore, the depletion of β-catenin in mouse endoderm produces multiple ectopic hearts, whereas inhibiting the Wnt signaling pathway in P19 cell culture models decreases cardiomyocyte differentiation [133,134]. Currently, the consensus on Wnt signaling is a biphasic role in cardiomyocyte differentiation, with the activation of Wnt signaling promoting mesoderm differentiation and the concomitant repression of Wnt signaling promoting cardiomyocyte differentiation [135,136,137]. Studies using mouse embryonic stem cells and zebrafish models reveal that the activation of Wnt signaling before gastrulation promotes cardiac differentiation. In contrast, continuous Wnt signaling activity during gastrulation inhibits cardiomyocyte formation [138,139]. Treating cells with Wnt3A-induced mesoderm differentiation and, in a feedback loop, represses the Wnt signaling pathway to promote cardiac differentiation [138]. 

The role of Wnt signaling in mesoderm differentiation is well established [137]. Earlier studies report the expression of Wnt3a before gastrulation in the proximal epiblast of the egg cylinder. Wnt3a expression is then limited to the posterior proximal epiblast and the visceral endoderm and is subsequently expressed in the primitive streak and mesoderm. The double knockout of Wnt3 in mouse embryos impairs mesoderm formation [140]. Moreover, mouse embryos lacking β-catenin cannot form mesoderm cells during gastrulation [141]. The induction of mesoderm cells via Wnt signaling has, therefore, been employed in several in vitro cardiomyocyte differentiation methods to generate high yields of cardiomyocytes [137,142,143,144]. Although Wnt signaling has been well studied in cardiomyocyte differentiation, significant gaps remain. For instance, the precise temporal regulation of the Wnt signaling pathway needs to be elucidated. There is also the need to resolve species-specific differences in the temporal regulation of Wnt signaling and differences between ESCs and iPSCs. It would also be interesting to study Wnt signaling in the context of other biphasic signaling pathways, such as the BMP signaling pathway, during cardiomyocyte differentiation. 

## 2. In Vitro Models for Cardiomyocyte Differentiation

**Figure 3:**
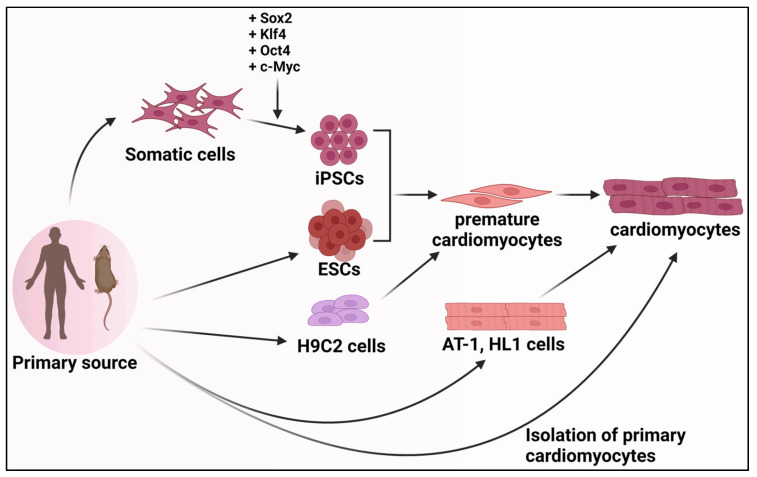
In vitro generation of cardiomyocytes. Illustration of the cardiomyocyte derivatization and generation from mouse and human sources. Cardiomyocytes can be derived directly from heart tissue and cultured in vitro or generated from many sources, including cardiomyocyte cell lines, embryonic stem cells, or induced pluripotent stem cells. Created with BioRender.com.

Studying the process of cardiomyocyte differentiation is critical to gaining insights into cardiac development, function, and disease. While studies can utilize organisms like mice and zebrafish as models for studying cardiomyocyte development and function, several cell culture-based methods exist to provide cheaper and easily adaptable alternatives. Among these are primary cardiomyocytes, embryonic stem cells, induced pluripotent stem cells, cardiac progenitor cells, and cardiomyocyte cell lines (Figure 3).

### 2.1. Primary Cardiomyocytes

Primary cardiomyocytes are directly isolated from heart tissues and are an excellent model for studying the complex processes that underlie the cellular basis of cardiac disease and function [145]. However, these cardiomyocytes cannot proliferate and are inadequate for studying the regulatory processes. The isolation of cardiomyocytes from fetal hearts is easy and more robust than adults, and they can survive longer ex vivo under specific culture conditions. The fetal cardiomyocytes can also be transfected with nonviral gene transfer methods and, therefore, can be used to study regulatory mechanisms [146,147,148]. Louch et al. describe standard methods to isolate and culture primary cardiomyocytes from neonatal and adult cardiomyocytes [149]. Primary cardiomyocytes are terminally differentiated and cannot be used to study the early stages of differentiation and the complexities associated with the genetic modification of these cells [145]. Consequently, alternative sources of cardiomyocytes are being explored to circumvent these challenges. 

### 2.2. Cardiomyocyte Cell Lines

Several attempts have been made to develop immortalized cardiomyocyte cell lines to investigate various aspects of cardiomyocyte biology. Currently, there are four cardiomyocyte cell lines, including AT-1, HL-1, and ANF-T-antigen, which are derived from mouse atrial cardiomyocyte tumors [150,151,152,153], and the widely used H9C2 cell line derived from embryonic rat ventricular tissue [154]. These cell lines exhibit hallmarks of cardiomyocytes, such as spontaneous contractile activity and the expression of cardiomyocyte-specific genes [145]. The H9C2 cell line closely resembles neonatal cardiomyocytes. It can be used extensively to study early development processes in cardiomyocytes. In contrast, the AT-1 and HL-1 cell lines possess a more differentiated phenotype and can be used to model adult cardiac diseases and for therapeutic drug monitoring [155]. A recent study utilizing H9C2 cells revealed that by maintaining mitochondria integrity, *Ohwia caudata* prevents doxorubicin-induced cardiotoxicity [156]. Additionally, H9C2 cells were used as a model for myocardial hypertrophy to discover the positive effects of the Cox inhibitors, aspirin, and celecoxib in protecting the heart from hypertrophy via their effects on the Notch1/Hes1 pathway [157]. 

For humans, the AC16 cardiomyocyte cell line, derived from non-proliferating primary cultures of adult ventricular heart tissue fused with SV40 transformed, uridine auxotroph human fibroblasts lacking mitochondria, is widely utilized as a model system for studying cardiomyocyte biology and disease modeling [158,159]. Recent studies have utilized AC16 cell lines to induce unfolded protein response (UPR), often occurring in stressed cells, which aided in the discovery of changes in the proteome, such as stress granule formation, membrane transporter localization, and possible endomembrane trafficking [160]. Similarly, AC16 cells were used as a model system to investigate the role of WNT signaling in protecting against cardiotoxicity, revealing a role via WNT10b in suppressing pro-apoptotic p38 and anti-apoptotic ERK1/2 activities [161].

A major advantage of using cardiomyocyte cell lines is their ease of maintenance and susceptibility to genetic modifications. While the cell lines can provide valuable information for cardiomyocyte development and disease, a caveat is that these cell lines do not resemble native adult cardiomyocytes, may not utilize the canonical differentiation signaling pathways, and may not fully recapitulate the complexity of primary cardiomyocytes or the native cardiac tissue environment [162]. 

### 2.3. Embryonic Stem Cells (ESCs) and Induced Pluripotent Stem Cells (iPSCs)

The use of ESCs and iPSCs has revolutionized the field of cardiac research, providing diverse opportunities to study early cardiomyocyte development. ESCs are derived from the inner cell mass of the blastocyst during early development, can be cultured almost indefinitely in vitro, and retain the ability to differentiate into any cell type of the mature organism. iPSCs, on the other hand, are generated directly from differentiated somatic cells by the introduction of key transcription factors, Oct4, Sox2, Klf4, and Nanog, which reverses the terminally differentiated cells into a pluripotent state capable of self-renewal and differentiation into the derivatives of all three germ layers, akin to ESCs. Using ESCs and iPSCs can recapitulate the differentiation of cells from pluripotency into cardiomyocytes, providing a unique model system to study early developmental processes. 

Since the heart is the first organ formed during mammalian development, the differentiation of stem cells into embryoid bodies, a three-dimensional aggregate of germ layer cells spontaneously generates beating cardiomyocytes without the addition of any external cytokines or drugs [163,164,165]. However, only a small percentage of cells in the embryoid body differentiate into cardiomyocytes [163]. To improve the yield of cardiomyocytes generated from embryoid bodies, methods, such as treatment with 5-azacytidine, a DNA demethylating agent, and differentiation under low oxygen tension, were developed [166,167]. However, these methods are still insufficient to generate cardiomyocytes at high yields and purity. Methods have been developed, often utilizing knowledge from normal cardiomyocyte differentiation in vivo, to generate cardiomyocytes efficiently and at high yields using stem cells. Some of these methods include the treatment of stem cells with Activin A, BMP4, bFGF, VEGF, and Dkk-1 to enhance the yield and purity of cardiomyocytes generated from stem cells [168,169,170,171]. 

The Wnt signaling pathway has become an attractive target for the efficient differentiation of stem cells into cardiomyocytes because of its established role in cardiomyogenesis and the development of inexpensive small molecules that inhibit or activate the Wnt pathway. Currently, two major variants of modulating WNT signaling for cardiomyocyte differentiation exist. The first variant only uses small molecule WNT pathway inhibitors during cardiomyocyte differentiation from stem cells. This method relies on the endogenous activation of WNT signaling and generates less than 60% of the yield of cardiomyocytes [172,173]. The second variant uses small molecules to activate and inhibit the WNT pathway, significantly increasing the yield of cardiomyocytes to over 85% [174,175,176,177]. 

ESCs provide several advantages for cardiomyocyte differentiation, including their robust ability to differentiate into cardiomyocytes, the wealth of resources and prior research on their utilization, and their electromagnetic binding to the host myocardium. However, there are serious ethical concerns around obtaining human ESCs, and cardiomyocytes generated from ESCs may face immune rejection if used for therapeutic purposes. In this regard, iPSCs provide several advantages, such as bypassing ethical concerns, being isolated from a diverse population of donors, and being patient-specific. However, the reprogramming of somatic cells into iPSCs can be variable and inefficient, creating challenges in reproducibility. iPSCs exhibit different genetic and epigenetic landscapes from their original somatic cells, potentially causing genome instability and affecting their functionalities [178,179]. 

## 3. Open Questions

Cardiovascular diseases are still the leading cause of mortality globally, with cardiomyocyte insufficiency underlying most heart failures. Knowledge of the development of cardiomyocytes is critical to unlocking intricate mechanisms that regulate growth and proliferation. Understanding these mechanisms will be a significant medical breakthrough, paving the way for personalized heart tissue regeneration and developing new therapeutic targets against heart failure. Despite extensive research in cardiomyocyte development, significant gaps remain. A widely accepted dogma for post-natal cardiomyocytes is their inability to proliferate. However, this notion has been highly contested with data supporting the presence of proliferating cardiomyocytes in the adult human heart [180]. Using the carbon-14 dating approach, Bergmann et al. predict that a small percentage of cardiomyocytes renew during the human lifespan, with a renewal of about 1% at the age of 25, which decreases to about 0.45% by age 75 [180]. However, their data do not conclude whether the cardiomyocyte renewal observed is from cardiomyocyte duplication or a stem cell niche in the heart [180]. In this regard, Senyo et al. used a combination of innovative approaches to show that newly derived cardiomyocytes originate from the division of pre-existing cardiomyocytes, especially at sites close to myocardial injury [181]. Similarly, the regenerative ability of cardiomyocytes was observed in one-day-old mice, with this ability lost by day 7 post-partum [182]. However, other studies also indicate the presence of cardiac stem cells as a source of new cardiomyocytes in adult mammalian hearts [183,184]. Regardless, major questions such as the extent of the cardiac repair processes, heterogeneity of cardiac cell populations, and paracrine effects of other cell types all add to the complexity of cardiomyocyte development. Further research is needed to reach a unanimous consensus on the post-natal proliferation of cardiomyocytes and the presence, isolation, and growth of cardiac stem cells for cardiac regenerative purposes. 

Using ESCs and iPSCs has contributed to several discoveries on cardiomyocyte development. However, there is considerable heterogeneity in the cell lines from ESCs and iPSCs and the differentiation protocols, including media composition, timing of differentiation, and culture conditions. Currently, cardiomyocytes are generated from ESCs and iPSCs using a myriad of methods, including the handing drop method in differentiation media without any supplements (yields the least amounts of cardiomyocytes by far), the growth of cardiomyocytes via monolayer cultures, the use of growth factors, and the use of small molecule inhibitors, especially those that modulate the WNT signaling pathway [185]. These differences in the generation of cardiomyocytes generate different yields of cardiomyocytes and could cause major differences in experimental conclusions regarding specific stages of cardiomyocyte development. Therefore, there is a need to optimize differentiation protocols to enhance the homogeneity of cardiomyocytes derived from iPSCs and ESCs. Additionally, the differentiation of ESCs and iPSCs into cardiomyocytes lacks the complexity of other cell types that make up the heart, thereby limiting knowledge on the in vivo interaction of cardiomyocytes with neighboring cells. 

The differentiation of cardiomyocytes during early development is a multiplex process integrating different signaling pathways with the gene expression of transcription factors that also regulate these signaling pathways. Most of these signaling pathways begin with binding signaling ligands that lead to a cascade of intracellular signaling events to turn on target genes. It becomes a “chicken and egg” conundrum regarding the timing of transcription factors and signaling pathways in embryonic cardiomyocyte development. The crosstalk of signaling pathways also adds to the complexity of cardiomyocyte development. Moreover, signaling pathways turn on several novel genes that may be essential to cardiomyocyte development. However, most studies focus on the core transcriptional network, which is widely studied during cardiomyocyte development, including Nkx2.5, Gata4, Mef2c, etc. With the advancement and accessibility of omics, including genomics, transcriptomics, and proteomics, researchers can study the role of transcription factors in the context of signaling events during cardiomyocyte differentiation. 

In conclusion, a holistic understanding of signaling pathways regulating cardiomyocyte differentiation holds immense therapeutic potential for cardiac regeneration and disease management. The targeted modulation of these signaling pathways will aid in the in vitro differentiation of cardiomyocytes and unravel molecular mechanisms underlying cardiomyocyte proliferation, survival, and maturation. Understanding these mechanisms will deepen our understanding of cardiac development and pave the way for innovative therapeutic strategies. 

## Data Availability

No new data were created or analyzed in this study.
